# Cerebrovascular response to an acute bout of low-volume high-intensity interval exercise and recovery in young healthy adults

**DOI:** 10.1152/japplphysiol.00484.2021

**Published:** 2021-12-09

**Authors:** Alicen A. Whitaker, Stacey E. Aaron, Carolyn S. Kaufman, Brady K. Kurtz, Stephen X. Bai, Eric D. Vidoni, Robert N. Montgomery, Sandra A. Billinger

**Affiliations:** ^1^Department of Physical Therapy, Rehabilitation Science, and Athletic Training, University of Kansas Medical Center, Kansas City, Kansas; ^2^Department of Molecular and Integrative Physiology, University of Kansas Medical Center, Kansas City, Kansas; ^3^Department of Physical Medicine and Rehabilitation, University of Kansas Medical Center, Kansas City, Kansas; ^4^University of Kansas Alzheimer’s Disease Research Center, Fairway, Kansas; ^5^Department of Biostatistics & Data Science, University of Kansas Medical Center, Kansas City, Kansas; ^6^Department of Neurology, University of Kansas Medical Center, Kansas City, Kansas

**Keywords:** blood flow, cerebral blood velocity, conductance, HIIE, high-intensity interval training

## Abstract

High-intensity interval exercise (HIIT) is performed widely. However, there is a gap in knowledge regarding the acute cerebrovascular response to low-volume HIIT. Our objective was to characterize the middle cerebral artery blood velocity (MCAv) response during an acute bout of low-volume HIIT in young healthy adults. We hypothesized that MCAv would decrease below the baseline (BL), *1*) during HIIT, *2*) immediately following HIIT, and *3*) 30 min after HIIT. As a secondary objective, we investigated sex differences in the MCAv response during HIIT. Twenty-four young healthy adults completed HIIT [12 males, age = 25 (SD = 2)]. HIIT included 10 min of 1-min high intensity (∼70% estimated maximal Watts) and active recovery (10% estimated maximal Watts) intervals on a recumbent stepper. MCAv, mean arterial pressure (MAP), heart rate (HR), and end-tidal carbon dioxide ($PET_{CO_{2}}$) were recorded at BL, during HIIT, immediately following HIIT, and 30 min after HIIT. Contrary to our hypothesis, MCAv remained above BL during HIIT. MCAv peaked at *minute 3* then decreased concomitantly with $PET_{CO_{2}}$. MCAv was lower than BL immediately following HIIT (*P* < 0.001). Thirty minutes after HIIT, MCAv returned to BL (*P* = 0.47). Compared with men, women had a higher MCAv at BL (*P* = 0.001), during HIIT (*P* = 0.009), immediately following HIIT (*P* = 0.004), and 30 min after HIIT (*P* = 0.001). MCAv did not decrease below BL during low-volume HIIT. However, MCAv decreased below BL immediately following HIIT and returned to resting values 30 min after HIIT. MCAv also differed between sexes.

**NEW & NOTEWORTHY** We are the first, to our knowledge, to characterize the cerebrovascular and hemodynamic response to low-volume high-intensity interval exercise (HIIT, 1-min intervals) in young healthy adults. Middle cerebral artery blood velocity (MCAv) decreased during the HIIT bout and rebounded during active recovery. Women demonstrated a significantly higher resting MCAv than men and the difference remained during HIIT. Here, we report a novel protocol and characterized the MCAv response during an acute bout of low-volume HIIT.

## Graphical abstract

**Figure:**
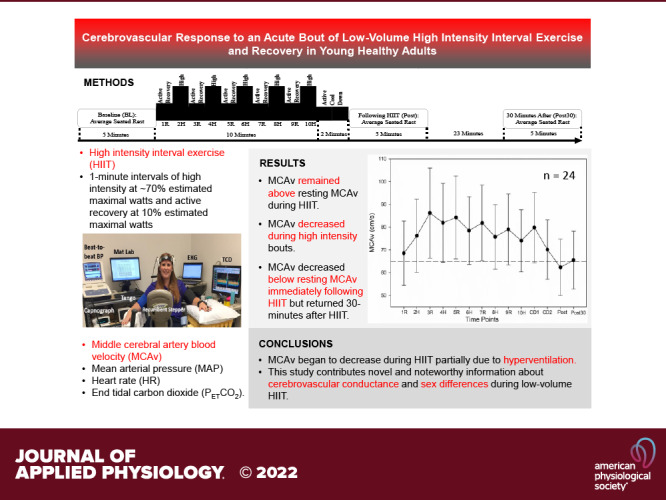

## INTRODUCTION

Temporal changes in cerebral blood flow, measured by middle cerebral artery blood velocity (MCAv), have been well characterized during submaximal continuous exercise (1–4). During steady-state low- and moderate-intensity continuous exercise, MCAv remains at a consistent rate (2, 5). As exercise intensifies, many physiological factors including cardiac output, sympathetic activity, blood pressure, and metabolism contribute to MCAv increasing in tandem up to anaerobic threshold (1, 6, 7). Beyond the anaerobic threshold, MCAv decreases with high-intensity exercise (1, 4, 6). One possible explanation of MCAv decreasing after the anaerobic threshold could be due to hyperventilation, resulting in hypocapnia or decreased partial pressure carbon dioxide ($Pa_{\text{CO}}{}_{{}_{2}}$) (8). To attenuate the decrease in $Pa_{\text{CO}}{}_{{}_{2}}$, downstream arteriole vasoconstriction occurs resulting in a reduced MCAv (9).

Although MCAv has been characterized during an acute bout of high-intensity continuous exercise (1, 3), our recent systematic review determined that few studies have examined MCAv during an acute bout of high-intensity interval exercise (HIIT) (10). HIIT is defined as repetitive short bouts of exercise switching between intervals of high intensity and recovery. Many different classifications exist of interval exercise including sprint interval exercise, high-volume HIIT, and low-volume HIIT (11–15). Sprint interval exercise includes short interval durations (8–30 s) at maximal workloads (>100%) (11). High-volume HIIT is characterized by longer duration intervals of 1–4 min with a cumulative time within high intensity ≥ 15 min (11). Although low-volume HIIT also has intervals of 1–4 min, the cumulative time of high intensity is < 15 min (11, 13, 14).

Previous studies have examined MCAv during sprint interval and high-volume HIIT in young healthy adults. During a single 30-s maximal sprint interval bout, MCAv initially increased during the sprint exercise followed by a decrease toward the baseline in young healthy women (∼ 27 yr of age) (16), and MCAv decreased below the baseline by 10% in young healthy men (∼25 yr old), suggesting that sex differences are present with maximal effort exercise (17). During an acute 20-min bout of sprint interval exercise in healthy young adults (∼23 yr of age), repetitive 30-s maximal effort sprint intervals (200% maximal Watts) separated by 4.5 min of active recovery caused MCAv to decrease during the second–fourth sprint bouts compared with the first sprint bout. However, MCAv did not decrease below baseline (BL) (18). The same study also investigated high-volume HIIT of four bouts of 4-min high intensity at 85% maximal heart rate and also reported a decrease in MCAv during the second–fourth bout with MCAv remaining above the BL (18). The longer recovery intervals of 3–4.5 min during sprint interval exercise and high-volume HIIT may have allowed for stabilization of $Pa_{\text{CO}}{}_{{}_{2}}$, therefore, maintaining MCAv above the baseline (18).

Interval exercise at moderate intensity has been proposed to be beneficial for cerebrovascular function in older men due to the active recovery intervals sustaining an elevated MCAv with recovery of expired end-tidal carbon dioxide ($PET_{CO_{2}}$) (19). However, sustained MCAv during HIIT has been stated to be counterintuitive due to decreases in $PET_{CO_{2}}$ (19). Although the 3–4.5 min of active recovery during sprint interval and high-volume HIIT may have allowed for recovery of $PET_{CO_{2}}$, low-volume HIIT with an active recovery of 1 min may not be enough time to recover $PET_{CO_{2}}$ (17). Decreasing $PET_{CO_{2}}$, therefore, may lead to further reductions of MCAv below the baseline, which was previously shown during an acute 12-min HIIT bout (20). Although this has been suggested, no studies have reported the effects of low-volume HIIT on MCAv in young healthy adults. HIIT has also been shown to attenuate peripheral vascular function immediately following and up to 1 h after exercise (13, 14, 21–23). Therefore, impaired vascular function following HIIT may also extend to the cerebrovascular system. Although MCAv recovery immediately following low-volume HIIT has not been previously studied in young healthy adults, previous work has shown reduced MCAv immediately following a HIIT exercise bout (20). To address the gap in cerebrovascular knowledge in young healthy adults, we proposed to characterize the MCAv response during a single bout of low-volume HIIT, with 1-min intervals of high intensity and active recovery, immediately after HIIT and at 30 min after HIIT.

Our primary hypothesis was that MCAv would significantly decrease below BL MCAv during an acute 10-min bout of low-volume HIIT and would be related to decreases in $PET_{CO_{2}}$. Our secondary hypotheses were that MCAv would be significantly lower than BL, *1*) during passive recovery immediately following HIIT and *2*) at rest, 30 min after HIIT.

Our previous work identified unique sex-specific trajectories in resting MCAv over the lifespan (24) and the MCAv response to moderate-intensity continuous exercise (25). Differences in resting MCAv may be driven by hormonal differences, peripheral resistance, blood pressure, and endothelial vasodilation (26–28). However, it is unknown whether there is a sex-specific cerebrovascular response to HIIT. As a secondary objective, we investigated whether sex differences existed in the MCAv response to low-volume HIIT. Based on our previous work, we hypothesized that women would have *1*) a higher absolute resting BL MCAv and *2*) a subsequently higher absolute MCAv response to HIIT compared with men.

## METHODS

Individuals were recruited from the surrounding community using flyers. Participants were enrolled in the study if they met the following inclusion criteria: *1*) 18–30 yr old and *2*) low cardiac risk defined by the American College of Sports Medicine (ACSM) (29). The study and all experimental procedures were approved by the University of Kansas Medical Center Human Subjects Committee. This study is registered on clinical trials.gov (NCT04673994). Before the start of study procedures, participants provided written informed consent.

### Study Visit 1

We collected demographic information including age, sex, race/ethnicity, body mass index (BMI), current medications, medical history, and self-reported physical activity within the past month via an exercise questionnaire (30, 31). Participants also completed the ACSM Risk Stratification Screening (32).

### TCD signal acquisition.

Following the questionnaires, participants were screened to ensure an MCAv signal on the right and/or left temporal window. Participants were fitted with an adjustable headband that secured bilateral transcranial Doppler ultrasound (TCD) probes (2-MHz, Multigon Industries, Inc., Yonkers, NY) with ultrasonic gel onto the temporal region of the participant’s head. MCAv signal was acquired using standard probe positioning, orientation, depth, and direction (33). The TCD depth, gain, power, and probe position were recorded on *study visit 1* for ease of signal acquisition on *study visit 2*.

### Submaximal exercise test.

Aligned with previous work, we chose a submaximal exercise test to determine the workload and heart rate range (19). Participants performed a submaximal exercise test on the total body recumbent stepper (TBRS) to determine the workload for an acute bout of interval exercise and to maintain the same mode of exercise used for HIIT bout. Our laboratory team developed the TBRS submaximal exercise test which was based on the established Young Men’s Christian Association (YMCA) protocol (34) and has been shown to be valid and reliable in young and older adults and in individuals with stroke (35–37). We fitted participants with a heart rate monitor (Polar Electro, Inc., NY) and then were seated on the TBRS (T5XR NuStep, Inc. Ann Arbor, MI). We performed the TBRS submaximal exercise test according to previously published methodology (35), with the exception that participants were instructed to perform exercise by only using their lower extremities. For the acute HIIT bout, participants would be exercising with their lower extremities since participants rested their arms on platforms at heart level to allow for accurate blood pressure measures. All participants began exercising at 30 W and stepped at a constant pace between 95–100 steps/ min. Every minute, heart rate (HR) and the rating of perceived exertion (RPE) were measured. Termination of the TBRS submaximal exercise test included: *1*) completion of all four stages of the exercise test, *2*) participant requested to stop the test, or *3*) participant reached 85% of their age-predicted maximal HR [(220 − age) × 0.85] (38). The TBRS submaximal exercise linear regression equation was then used to calculate the estimated maximal oxygen consumption (estimatedV̇o_2max_) (35). Based on the YMCA protocol, the linear relationship between workload and HR (i.e., slope) was plotted and used to determine the estimated maximal watts (estimatedWatt_max_) (34), and then we calculated the exercise intensity dose (Watts) for the high intensity and active recovery intervals.

### Exercise familiarization.

After the TBRS submaximal exercise test, participants rested to allow HR and blood pressure to return to resting values. Participants were fitted with a nasal cannula and wore it during the HIIT familiarization to practice breathing through their nose. Individuals participated in the HIIT familiarization, switching between 1-min high intensity and 1-min active recovery while maintaining a consistent step rate on the recumbent stepper. Based on previous HIIT protocols using peak power output, the intensity for the acute HIIT bout was calculated as ∼70% estimatedWatt_max_ for high intensity and 10% estimatedWatt_max_ for active recovery (13–15, 39–42). HR and RPE were measured at the end of each minute. Based on previous recumbent stepper HIIT protocols, a HR limit of 85% of age predicted maximum HR was used. The familiarization session lasted ∼10 min.

### Study Visit 2

#### Carotid ultrasound.

Upon arrival to the laboratory, participants underwent a carotid ultrasound scan. Participants rested quietly in a supine position for 20 min. We then conducted a bilateral Doppler ultrasound scan of the common, internal, and external carotid artery. Carotid two-dimensional (2-D; GE Healthcare LOGIQ Ultrasound, Chicago, IL) images as well as systolic and diastolic velocity measures were sent to a collaborating physician (S.X.B.) for carotid stenosis assessment (43).

#### MCAv recordings.

After completing the carotid ultrasound scan, participants were seated on the recumbent stepper and rested quietly for 20 min while the following equipment was donned: *1*) TCD headset and bilateral ultrasound probes to measure MCAv, *2*) a 5-lead electrocardiogram (ECG; Cardiocard, Nasiff Associates, Central Square, NY) for HR, *3*) a nasal cannula attached to a capnograph (BCI Capnocheck Sleep 9004 Smiths Medical, Dublin, OH) for $PET_{CO_{2}}$ and respiratory rate (RR), *4*) a beat-to-beat blood pressure cuff on the left middle finger (Finometer, Finapres Medical Systems, Amsterdam, The Netherlands) for mean arterial pressure (MAP), and *5*) a brachial automated sphygmomanometer with a microphone on the right arm (Tango M2; SunTech, Morrisville, NC) to ensure an accurate calibration of the Finometer. The participant’s arms were placed on stable platforms at heart level. To examine the conductance of peripheral blood pressure to the brain via MCAv, we calculated the beat-to-beat cerebrovascular conductance index (CVCi = MCAv/MAP) (16).

**Figure 1.**
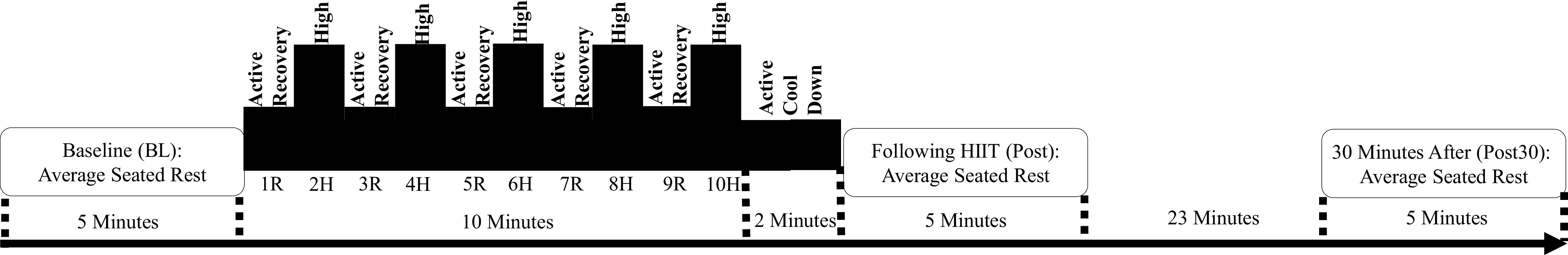
Protocol layout of MCAv recordings *1*) before HIIT, *2*) during HIIT, *3*) immediately following HIIT, and *4*) 30 min after the HIIT. BL, baseline; H, high intensity; HIIT, high-intensity interval exercise; MCAv, middle cerebral artery blood velocity; post, immediately following HIIT; post30, 30 min after HIIT; R, active recovery.

As in our previous work, the laboratory room was dimly lit and kept at a constant temperature of 22°C–24°C (5). Figure 1 details the experimental protocol. Following BL rest recording, MCAv was continuously recorded during the 10-min acute HIIT bout. The HIIT bout consisted of repetitive 1-min high intensity (∼70% estimatedWatt_max_) separated by 1-min active recovery (10% estimatedWatt_max_) (13–15, 19, 39–42). The HIIT MCAv recording started with a 1-min active recovery interval to decrease the resistance of inertia before high intensity and thus prevent a Valsalva maneuver, which can elicit a large transient drop in MAP and MCAv (44). After 10 min of HIIT, resistance was decreased to 15 W and participants performed a 2-min active cool down. Immediately following the cool down, participants stopped the exercise and sat quietly on the recumbent stepper while we collected a 5-min recording. Participants remained seated on the recumbent stepper and the final 5-min seated rest recording was conducted at 30 min after HIIT.

Raw data were acquired through an analog-to-digital unit (NI-USB-6212, National Instruments) and custom-written software within MATLAB (v2014a, The MathWorks, Inc., Natick, MA). Sampling rate was set at 500 Hz. Raw data were then time aligned and interpolated beat-to-beat using the R spike on ECG as a trigger. The seated rest recordings at BL, immediately following HIIT, and 30 min after HIIT, were calculated as a 5-min average. The 10-min HIIT recording was separated into 1-min averages of each high intensity and active recovery bout. Consistent with previously published work, the left MCAv signal was used within the analysis to compare time points but if the left MCAv signal was not obtainable, then right MCAv was used (5).

### Data Analysis

The primary aim of this study was to determine whether MCAv decreased below BL during HIIT. To analyze this aim, we used a repeated measures MANOVA and conducted Bonferroni adjusted post hoc paired *t* tests. The assumptions of normality and sphericity were checked using a Shapiro–Wilk test and Mauchly’s test, respectively. The secondary aims comparing MCAv to baseline immediately following HIIT and 30-min after HIIT were analyzed identically. Compared with BL, a repeated-measures MANOVA was performed on the secondary dependent variables, MAP, HR, $PET_{CO_{2}}$, and CVCi during HIIT. To evaluate the effect of sex while adjusting for time, our primary analysis used a mixed ANOVA with a within subjects’ effect for time and a between subjects’ effect for sex. In addition, we performed a secondary analysis of post hoc *t* tests for MCAv between sexes conducted at every time point. The relationships between MCAv, MAP, and $PET_{CO_{2}}$ were pooled from change scores calculated for each participant and analyzed using Spearman’s rank correlation coefficients. Participant characteristics were analyzed using a one-way ANOVA, Mann–Whitney *U* test for nonparametric variables, and Fisher’s exact test for categorical variables.

## RESULTS

Twenty-five young healthy adults (12 women, 13 men) completed the study consisting of two visits to the laboratory. The second visit occurred 48 h to 2 wk after the initial visit. No adverse events occurred. Due to the loss of TCD signal during HIIT, we excluded one male individual from the data analysis. Due to loss of capnograph signal during HIIT, one female individual did not have a valid $PET_{CO_{2}}$ and RR recording during HIIT. Three participants required rescheduling for *study visit 2* due to campus-wide closure for weather. These individuals completed *study visit 2* within 1 mo of the initial study visit. All participants confirmed that they refrained from vigorous exercise for 24 h (45) and from consuming caffeine for 8 h (46–48) and alcohol for 24 h (49). All participants reported no change in health or physical activity levels between study visits. For women, visits were scheduled between *days 1* and *7* of the menstrual cycle (50). Study visits were scheduled between 2 and 5 PM to maintain consistency and minimize circadian influence on cerebral blood velocity (51). Participant characteristics are presented in Table 1. All participants were classified as having <50% carotid stenosis (43). Seven out of twelve (58%) women reported using birth control medication. During the acute exercise bout, all participants maintained the prescribed workload during all bouts of high intensity and active recovery.

**Table 1.** Participant characteristics

	All	Women	Men	*P* Value
*n*	24	12	12	
Age, yr	25 (SD 2)	25 (SD 2)	25 (SD 1)	0.93
BMI, kg/m^2^	23.7 (SD 3.8)	21.6 (SD 4.0)	25.8 (SD 2.2)	0.004*
Race/ethnicity, *n* (%)				
White/Caucasian	17 (71%)	9 (75%)	8 (67%)	1.00
Hispanic/Latino	1 (4%)	0	1 (8%)	1.00
Asian	6 (25%)	3 (25%)	3 (25%)	1.00
Native American	1 (4%)	0	1 (8%)	1.00
Physical activity score, *n* (%)				
<10 min, 5 times/wk	1 (4%)	1 (8%)	0	
20–60 min/wk	2 (8%)	1 (8%)	1 (8%)	
1–3 h/wk	2 (8%)	1 (8%)	1 (8%)	
>3 h/wk	19 (79%)	9 (75%)	10 (83%)	1.00
Estimated V̇O_2max_, mL·kg^−1^·min^−1^	44.7 (SD 6.8)	41.4 (SD 4.4)	48.0 (SD 7.4)	0.02*
Regularly performs HIIE, *n* (%)	11 (46%)	7 (58%)	4 (33%)	0.41
Absolute high intensity resistance, W	134 (SD 37)	112 (SD 17)	157 (SD 38)	0.002*
Relative high intensity resistance (estimatedWatt_max_)	71% (SD 5%)	72% (SD 5%)	70% (SD 5%)	0.29
Absolute active recovery resistance, W	20 (SD 5)	17 (SD 2)	23 (SD 5)	<0.001*
Relative active recovery resistance (estimatedWatt_max_)	10% (SD 1%)	11% (SD 2%)	10% (SD 0.2%)	0.63
Average steps/min	99 (SD 2)	99 (SD 2)	99 (SD 2)	0.48

Means (standard deviations). *Significantly different between men and women (*P* < 0.05). One individual identified as multiracial. BMI, body mass index; estimatedWatt_max_, estimated maximal watts; HIIT, high intensity interval exercise; mL·kg^−1^·min^−1^, milliliters of oxygen per kilogram of body weight per minute; W, watts. V̇o_2max_, maximal oxygen consumption.

### MCAv Response to HIIT

**Figure 2.**
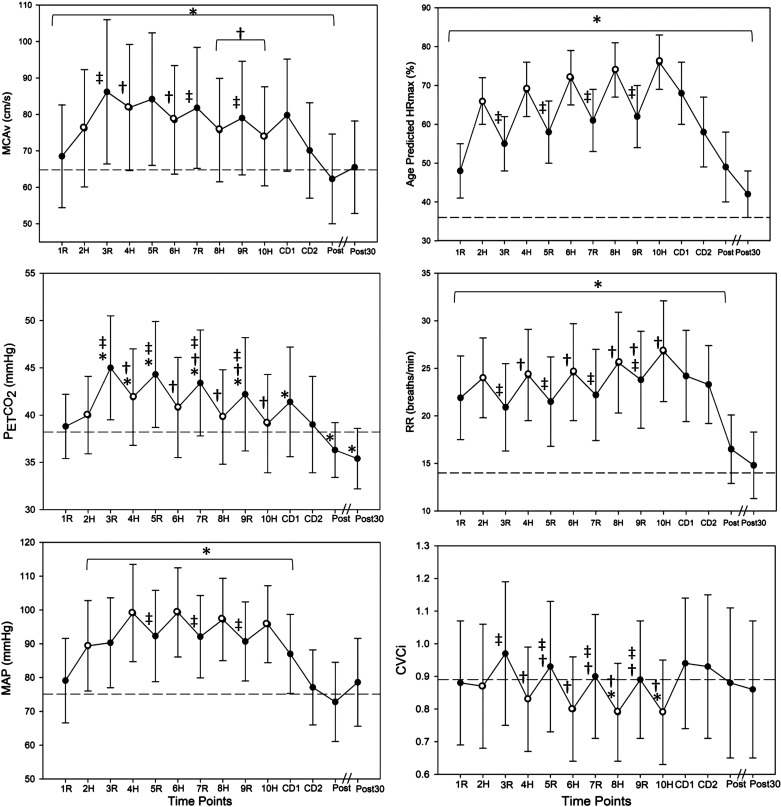
Response to high-intensity interval exercise (HIIT, *n* = 24); horizontal dashed line represents baseline value. *Significantly different than baseline (*P* < 0.005). †Significantly different than *minute 3* (MCAv peak). ‡Significantly different than previous high-intensity bout. Middle cerebral artery blood velocity (MCAv, cm·s^−1^), age-predicted maximal heart rate (HRmax), expired end-tidal carbon dioxide ($PET_{CO_{2}}$, mmHg, *n* = 23), respiratory rate (RR, breaths/min, *n* = 23), mean arterial pressure (MAP, mmHg), and cerebrovascular conductance index (CVCi, MCAv/MAP). CD, cool down; H, high intensity (open dots); post, immediately following HIIT; post30, 30 min after HIIT; R, active recovery.

The average MCAv for each minute of HIIT is presented in Fig. 2. Odd numbered minutes represent active recovery while even numbered minutes (open dots) represent high intensity exercise. Compared with the BL, MCAv during HIIT was not lower (*P* > 0.99). In fact, MCAv was higher than BL during *minutes 1* through *10* of HIIT (*P* ≤ 0.003). MCAv peaked at *minute 3* and began to decrease during high intensity throughout the remainder of the acute exercise bout. We observed MCAv reach its peak at *minute 3* followed by a significantly lower response at *minutes 4*, *6*, *8*, *9*, and *10* (*P* ≤ 0.001). Following high intensity, we observed a “rebounding” response whereby MCAv increased significantly during active recovery *minute 3* (*P* < 0.001), *minute 7* (*P* = 0.001), and *minute 9* (*P* < 0.001). Compared with the BL, MCAv was also lower immediately following HIIT (*P* < 0.001) and returned to BL values at 30 min after HIIT (*P* = 0.47).

### MAP, HR, $PET_{CO_{2}}$, and CVCi Response to HIIT

During HIIT, MAP increased significantly from BL values (*P* < 0.001), shown in Fig. 2. MAP also significantly increased during high intensity compared with active recovery (*P* < 0.001). The change in MAP was significantly related to the change in MCAv between: *1*) BL and *minute 1* (*r* = 0.48, *P* = 0.02), *2*) *minutes 1* and *2* (*r* = 0.41, *P* = 0.05), *3*) *minutes 2* and *3* (*r* = 0.72, *P* < 0.001), *4*) *minutes 6* and *7* (*r* = 0.42, *P* = 0.04), and *5*) *minutes 8* and *9* (*r* = 0.45, *P* = 0.03). MAP returned to resting values and was not significantly different from BL, immediately following HIIT (*P* = 0.25) or 30 min after HIIT (*P* = 0.12).

Compared with the BL, $PET_{CO_{2}}$ increased significantly during HIIT (*P* < 0.001, *n* = 23), specifically during active recovery *minutes 3*, *5*, *7*, and *9* (*P* ≤ 0.002) and high-intensity *minute 4* (*P* < 0.001). Compared with BL, RR increased during HIIT (*P* < 0.001, *n* = 23) and was increased during high-intensity *minutes 2*, *4*, *6*, and *8* (*P* ≤ 0.003) compared with active recovery. Compared with *minute 3* (peak MCAv), RR increased during high-intensity *minutes 4*, *6*, *8*, and *10* and active recovery *minute 9* (*P* < 0.001), which is consistent with the pattern of hyperventilation- and hypocapnia-induced decreases in MCAv, shown in Fig. 2. Due to increased RR (hyperventilation) during high intensity, $PET_{CO_{2}}$ was not significantly different from BL during high-intensity *minutes 6*, *8*, and *10*. The change in $PET_{CO_{2}}$ was significantly related to the change in MCAv between BL and *minute 1* (*r* = 0.47, *P* = 0.02), *minutes 1* and *2* (*r* = 0.60, *P* = 0.002), *minutes 4* and *5* (*r* = 0.58, *P* = 0.004), *minutes 5* and *6* (*r* = 0.44, *P* = 0.04), *minutes 6* and *7* (*r* = 0.57, *P* = 0.005), and *minutes 7* and *8* (*r* = 0.61, *P* = 0.002). During recovery, $PET_{CO_{2}}$ was significantly lower than BL immediately following HIIT (*P* = 0.001) and 30 min after HIIT (*P* < 0.001). RR was lower than BL immediately following HIIT (*P* < 0.001) but returned to BL levels at 30 min after HIIT (*P* = 0.27).

**Figure 3.**
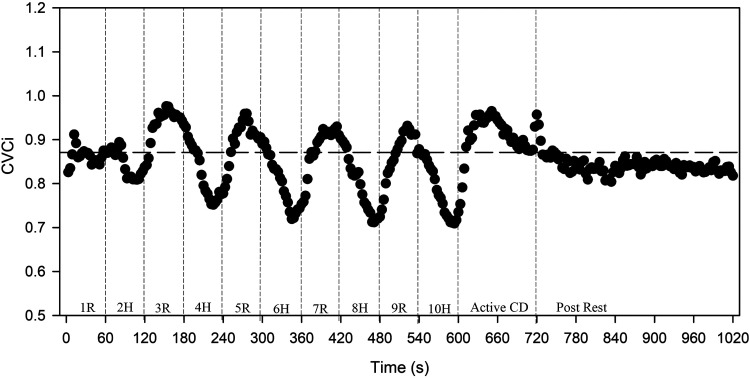
Cerebrovascular conductance index (CVCi, MCAv/MAP). Horizontal dashed line represents baseline value. High-intensity interval exercise (HIIT) occurred during 0–600 s (10 min). Active cool down occurred between 600 and 720 s (2 min). Passive recovery immediately following HIIT occurred between 720 and 1,020 s (5 min). CD, cool down; H, high intensity; MAP, mean arterial pressure; MCAv, middle cerebral artery blood velocity; post, immediately following HIIT; R, active recovery.

CVCi significantly decreased below BL during HIIT, specifically high-intensity *minutes 8* and *10* (*P* ≤ 0.004). CVCi returned to BL resting values immediately following HIIT (*P* = 0.71) and 30 min after HIIT (*P* = 0.28). Following *minute 3* (peak MCAv), CVCi decreased during high intensity and increased with active recovery (*P* < 0.001), shown in Fig. 3. The slope from the onset of each minute (high intensity or active recovery) to the peak CVCi was calculated for *minutes 1*, *2*, *3*, *5*, *7*, and *9*. With CVCi decreasing during *minutes 4*, *6*, *8*, and *10*, the slope from onset to nadir CVCi value was calculated. The slopes of the CVCi response were different between high intensity and active recovery following *minute 3* (*P* < 0.001). The first high-intensity bout, or *minute 2*, was faster to peak CVCi [19.1 (SD = 13.8) s] compared with other high-intensity intervals (*P* < 0.001). Following *minute 3*, the time to peak or nadir CVCi was also significantly faster during active recovery compared with high intensity such as *minutes 4* and *5* [$\overset{-}{x}$ = 41.7 (SD = 6.6) s vs. $\overset{-}{x}$ = 35.9 (SD = 9.1) s, *P* = 0.006], and *minutes 8* and *9* [$\overset{-}{x}$ = 47.2 (SD = 6.3) s vs. $\overset{-}{x}$ = 38.7 (SD = 11.0) s, *P* = 0.002].

### Sex Differences in MCAv Response to HIIT

**Figure 4.**
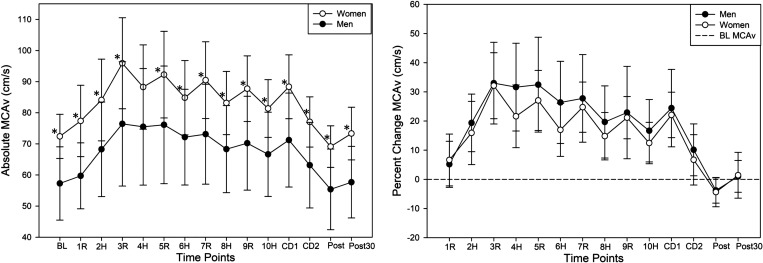
Absolute middle cerebral artery blood velocity (MCAv) response to high-intensity interval exercise (*left*) compared with the relative percent change in MCAv response (*right*). *Significantly different between men (*n* = 12) and women *(n* = 12). Horizontal dashed line represents baseline value. BL, baseline; CD, cool down; H, high intensity; post, immediately following HIIT; post30, 30 min after HIIT; R, active recovery.

The MCAv response to HIIT was significantly different between men and women (*P* = 0.009), shown in Fig. 4. MCAv was significantly higher in women compared with men at BL (*P* = 0.001) and during HIIT (*P* ≤ 0.03) except for high-intensity *minute 4* (*P* = 0.07). Although not significant, the percentage change in MCAv was visually higher in men compared with women during HIIT *minutes 2* through *10*, shown in Fig. 4. Although absolute workload was significantly higher in men compared with women (*P* = 0.002), the percent age-predicted HRmax response was significantly higher in women compared with men during HIIT (*P* = 0.009). MAP (*P* = 0.22) and $PET_{CO_{2}}$ (*P* = 0.18) were not significantly different between sexes during HIIT. Immediately following HIIT and 30 min after HIIT, MCAv remained significantly higher in women compared with men (*P* ≤ 0.004).

## DISCUSSION

Low-volume HIIT is widely used to improve aerobic fitness and peripheral vascular health (11, 52–54) despite minimal research into its effects on the cerebrovascular system (10). This study addresses a gap in knowledge and provides novel and critical insight into the MCAv response in young healthy adults to rapid and repetitive 1-min high-intensity and active recovery intervals of low-volume HIIT for 10 min. The primary findings of the study are *1*) MCAv did not decrease below resting values during HIIT; *2*) MCAv peaked within 3 min of HIIT and then began to decrease from the peak and was significantly lower at *minutes 4*, *6*, *8*, *9*, and *10*; *3*) MCAv rebounded during active recovery; *4*) MCAv decreased below resting BL values immediately following low-volume HIIT; and *5*) MCAv was significantly higher in women compared with men at BL, during HIIT, and up to 30 min after HIIT.

The primary objective of this study was to characterize the MCAv response during an acute bout of 1-min interval low-volume HIIT in young healthy adults. Based on previously published work (20), we hypothesized that MCAv would decrease below BL MCAv during an acute 10-min HIIT bout. However, our primary hypothesis was not supported by our findings. Although MCAv did not decrease below resting MCAv throughout the HIIT bout, MCAv did start to decrease after 3 min of HIIT. Initially, MCAv increased and the change in MCAv was moderately correlated with the change in MAP at the onset of HIIT. The change in MCAv was also moderately correlated to the change in MAP during the “rebounding” of active recovery from *minutes 6* to *7* and *minutes 8* to *9*. The change in MCAv was also moderately correlated to the change in $PET_{CO_{2}}$ at the onset of HIIT. Between *minutes 4* through *8* of HIIT, MCAv began decreasing with hyperventilation and the change in MCAv was moderately correlated with the change in $PET_{CO_{2}}$. Therefore, the initial rise in MCAv could be partially explained by peripheral blood pressure and increases in carbon dioxide while the latter decrease in MCAv could have been partially explained by hyperventilation- and hypocapnia-induced arteriole vasoconstriction. Although not measured within this study, exercise increases cardiac output and neuronal activity thereby elevating blood flow and shear stress, which may stimulate endothelial activity (55). We acknowledge that other physiological factors could have contributed to the MCAv response to HIIT such as metabolism, sex hormones, and sympathetic nervous system activity (1).

Prior research has demonstrated a decrease in MCAv below BL resting MCAv during 1-min interval low-volume HIIT in young children (20). In contrast, we did not find a reduction in MCAv below BL MCAv, which could be due to the age of the participants (children vs. adults), the high-intensity workload (90% vs. 70% estimated Watt maximum), and the exercise modality (cycle vs. recumbent stepper). Our results are supported by previous research performing short sprint interval training in young healthy adults that also found no reduction in MCAv below BL resting MCAv (18). However, the prior study performed 30-s sprint intervals at 200% W maximum with longer 4.5-min recovery intervals on a cycle. To our knowledge, we are also the first to characterize MCAv during HIIT using a recumbent stepper. We have previously published on the MCAv response to continuous exercise in young healthy adults, aging older adults, and clinical populations (5, 25, 56). The recumbent stepper has many benefits, such as the adaptive qualities for aging adults and clinical populations, and allows us to examine the MCAv response in all individuals. Although no studies have compared the MCAv response between recumbent stepper and other modes of exercise, previous studies comparing cycle ergometer and treadmill have reported differing MCAv responses, potentially due to pulse pressure oscillations (57). Therefore, the recumbent stepper MCAv response could potentially differ from other modes of exercise due to the stepping pattern and reclined posture. Our results show an increase in MCAv above the baseline during the high-intensity (65%–75% HRmax) bouts of low-volume HIIT on a recumbent stepper, which is consistent with previous research performing high-volume HIIT at 85% HRmax on a cycle (18). Our protocol demonstrated a consistent increase in MCAv relative to BL across all five high-intensity efforts including a rebound in MCAv during active recovery. Therefore, the short 1-min intervals of high intensity followed by active recovery may represent increased effects of shear stress stimulus and may be a promising exercise protocol for improving cerebrovascular health. However, more research is needed before definitive recommendations can be stated.

Although MCAv started to decrease during high intensity after 3 min of HIIT, MCAv increased or “rebounded” during active recovery. Previous studies measuring MCAv during HIIT and sprint interval exercise have also reported this “rebounding” effect (16, 18, 20). MCAv “rebounding” during active recovery could be partially explained by increases in $PET_{CO_{2}}$ as they were moderately correlated. MCAv also closely followed the pattern of $PET_{CO_{2}}$, with both measures decreasing during high intensity (hyperventilation) and increasing during active recovery. Others have hypothesized that increased shear stress on the cerebrovascular arterial walls with high-intensity exercise could create vasodilatory effects (18, 58). However, future research measuring dynamic cerebrovascular autoregulation and the cerebrovascular flow-mediated dilatory response to low-volume HIIT requires further exploration.

Our secondary hypotheses were that MCAv would be lower than BL MCAv during passive recovery *1*) immediately following HIIT and *2*) 30 min after HIIT. Our hypotheses were partially supported by our findings. MCAv was lower during the 5-min rest immediately following HIIT but not at 30 min after HIIT. Our findings are supported by previous research also reporting a reduction in MCAv below BL immediately following low-volume HIIT (20). However, the reduction in MCAv cannot be fully explained by $PET_{CO_{2}}$ considering MCAv recovers to BL resting values at 30 min after HIIT, whereas $PET_{CO_{2}}$ remains significantly decreased (20). This could be due to an attenuation of cerebrovascular reactivity to carbon dioxide, blood pressure, cardiac output, or other factors following an acute bout of HIIT (1, 10, 59).

### CVCi Response

We are the first, to our knowledge, to characterize the dynamic CVCi response during low-volume HIIT. Similar to Labrecque et al. (16) examining the time to peak MCAv during a 30-s high-intensity sprint, we characterized the time to peak (or nadir) CVCi during each 1-min high intensity and active recovery. We found that the time to peak CVCi was fastest during the first high-intensity interval compared with the remaining high-intensity intervals. We hypothesize that the longer time to peak/nadir CVCi during high-intensity intervals could be a regulatory response, attenuating quick fluctuations in CVCi with increased MAP. The slope of the CVCi response was negative during the latter high-intensity intervals, due to hyperventilation-induced decreases in MCAv. However, CVCi increased or “rebounded” during active recovery. Although prior work has shown a decreased regulatory response following HIIT (45), further research is needed to determine whether the increase in CVCi or “rebounding effect” is due to an attenuation of the regulatory response during the HIIT.

### Sex Differences

Our results are the first, to our knowledge, to show a significant difference in the MCAv response to low-volume HIIT between men and women. Our work has consistently identified women as having a higher BL resting MCAv compared with men (24, 25). However, we are the first to report that women may have a higher absolute MCAv response to low-volume HIIT compared with men. Previous work performing sprint interval exercise and high-volume HIIT reported no sex differences (18). As observed in Table 1, the men worked at a significantly greater absolute workload than women, which we propose influenced the relative MCAv response via the exercising skeletal muscle pressor reflex inducing hemodynamic changes (60). The MCAv differences between sexes could also be due to young men having a higher resting pulsatility index, as lower MCAv has been hypothesized to be a protective mechanism for the downstream microvasculature (24, 61). Another possible explanation for finding differences in the MCAv response to HIIT between sexes could be oral contraceptive use, enhancing the carbon dioxide chemoreflex (62, 63) and women having a greater reactivity to carbon dioxide (64). Although we standardized the visits to be within *days 1–7* of the follicular phase, we did not directly measure the effects of hormones on MCAv in both women and men in this study.

### Limitations

There are limitations for the current study. First, due to the difficulty of temporally assessing middle cerebral artery (MCA) diameter during high-intensity exercise, it was not measured during this study. There is much controversy as to how much the MCA diameter may change with a specific exercise stimulus (65–68). However, MCAv is a separate measure to cerebral blood flow and TCD is currently the best method to examine the cerebrovascular response to exercise with a high temporal resolution (65). Second, our HIIT protocol was based on an estimated maximal intensity, rather than directly measuring the anerobic threshold from a maximal exercise test (12). We acknowledge that exercising at a higher intensity could have caused $PET_{CO_{2}}$ and MCAv to decrease at a greater extent than reported here. However, our findings show that the $PET_{CO_{2}}$ response decreased continuously after *minute 3* (see Fig. 2) during the HIIT bout. Third, three individuals were also outside the window of 2–14 days between study visits. However, these individuals confirmed no changes to their health or physical activity routine between *study visits 1* and *2*. Therefore, in the context of this study design in young healthy adults, the results were not likely skewed. Last, $PET_{CO_{2}}$ was measured via nasal cannula, and breathing strictly through the nose during HIIT could become increasingly difficult. A study team member monitored nasal breathing throughout the entirety of the MCAv recordings and acute HIIT bout to ensure adherence to the protocol and appropriate $PET_{CO_{2}}$ values. Although not measured, nasal breathing could have artificially led to accumulation of $Pa_{\text{CO}}{}_{{}_{2}}$ and an elevated MCAv throughout the exercise (69).

### Future Considerations

Prior literature reviews suggest HIIT results in rapid changes (oscillations) in blood pressure that could challenge the cerebrovascular system, particularly those with cerebrovascular impairment (55). Despite these proposed considerations, high-intensity gait training and interval exercise are recommended to improve walking for those with neurological disease (70). Furthermore, clinical practice guidelines are beginning to include HIIT to improve walking outcomes after stroke (39, 70, 71). We are currently participating in a multisite R01 and have published a locomotor HIIT protocol in individuals with stroke performing 1-min intervals of high intensity and recovery (72). Therefore, the next logical step in this cerebrovascular research will focus on studying low-volume HIIT with 1-min intervals in individuals with stroke.

### Conclusions

During an acute 10-min bout of low-volume HIIT, MCAv does not decrease below BL resting MCAv in young healthy adults. However, MCAv decreased below BL resting MCAv immediately following HIIT but returned to BL at 30 min after HIIT. Further exploration into the regulatory response and physiological mechanisms contributing to a reduction in MCAv during passive recovery after HIIT is needed. Changes to the cerebrovascular response after a long-term exercise intervention of low-volume HIIT is also needed to address the gaps in knowledge on chronic cerebrovascular adaptations. This study contributes novel characterizations of the MCAv response to low-volume HIIT in young healthy adult controls, including changes in CVCi and sex differences. Although previous studies have provided groundwork on the MCAv response to sprint interval exercise and high-volume HIIT (16, 18), we have contributed to the existing literature studying the cerebrovascular response to low-volume HIIT in healthy young adults and the ability to extend this work to older adults and clinical populations such as stroke.

## GRANTS

A.W. was supported by the Eunice Kennedy Shriver National Institute of Child Health & Human Development of the National Institutes of Health Grant T32HD057850. S.E.A. was supported by a CTSA Grant from NCATS awarded to the University of Kansas for Frontiers: University of Kansas Clinical and Translational Science Institute Grant TL1TR002368. S.A.B. and E.D.V. were supported in part by the KU Alzheimer's Disease Research Center (P30 AG072973) from the National Institute on Aging. REDCap at University of Kansas Medical Center is supported by Clinical and Translational Science Awards (CTSA) Award No. UL1TR000001 from National Center for Research Resources (NCRR). The Georgia Holland Research in Exercise and Cardiovascular Health (REACH) laboratory space was supported by the Georgia Holland Endowment Fund.

## DISCLAIMERS

The content is solely the responsibility of the authors and does not necessarily represent the official views of the National Institutes of Health.

## DISCLOSURES

No conflicts of interest, financial or otherwise, are declared by the authors.

## AUTHOR CONTRIBUTIONS

A.A.W., E.D.V., and S.A.B. conceived and designed research; A.A.W. and B.K.K. performed experiments; A.A.W., S.E.A., C.S.K., B.K.K., S.X.B., R.N.M., and S.A.B. analyzed data; A.A.W., S.E.A., C.S.K., and S.A.B. interpreted results of experiments; A.A.W. prepared figures; A.A.W., S.E.A., C.S.K., and S.A.B. drafted manuscript; A.A.W., S.E.A., C.S.K., B.K.K., S.X.B., E.D.V., R.N.M., and S.A.B. edited and revised manuscript; A.A.W., S.E.A., C.S.K., B.K.K., S.X.B., E.D.V., R.N.M., and S.A.B. approved final version of manuscript.
